# New Atg9 Phosphorylation Sites Regulate Autophagic Trafficking in Glia

**DOI:** 10.1080/17590914.2024.2443442

**Published:** 2025-01-14

**Authors:** Linfang Wang, Shuanglong Yi, Shiping Zhang, Yu-Ting Tsai, Yi-Hsuan Cheng, Yu-Tung Lin, Chia-Ching Lin, Yi-Hua Lee, Honglei Wang, Margaret S. Ho

**Affiliations:** a School of Life Science and Technology, ShanghaiTech University, Shanghai, China; b Institute of Neuroscience, National Yang Ming Chiao Tung University, Taipei, Taiwan; c Brain Research Center, National Yang Ming Chiao Tung University, Taipei, Taiwan; d The Institute of Seed Industry, Xianghu Laboratory, Qiantang River International Innovation Belt of the Xiaoshan Economic and Technological Development Zone, Hangzhou, China; e Shanghai Institute of Thoracic Oncology, Shanghai Chest Hospital, Shanghai Jiao Tong University School of Medicine, Shanghai, China; f State Key Laboratory of Medical Neurobiology and MOE Frontiers Center for Brain Science, School of Life Sciences, Fudan University, Shanghai, China; g The Key Laboratory of Developmental Genes and Human Disease, School of Life Science and Technology, Southeast University, Nanjing, China

**Keywords:** Atg9, Atg1, dAuxilin, glia, Parkinson’s disease

## Abstract

We previously identified a role for dAuxilin (dAux), the fly homolog of Cyclin G-associated kinase, in glial autophagy contributing to Parkinson’s disease (PD). To further dissect the mechanism, we present evidence here that lack of glial dAux enhanced the phosphorylation of the autophagy-related protein Atg9 at two newly identified threonine residues, T62 and T69. The enhanced Atg9 phosphorylation in the absence of dAux promotes autophagosome formation and Atg9 trafficking to the autophagosomes in glia. Whereas the expression of the non-phosphorylatable Atg9 variants suppresses the lack of dAux-induced increase in both autophagosome formation and Atg9 trafficking to autophagosome, the expression of the phosphomimetic Atg9 variants restores the lack of Atg1-induced decrease in both events. In relation to pathophysiology, Atg9 phosphorylation at T62 and T69 contributes to dopaminergic neurodegeneration and locomotor dysfunction in a *Drosophila* PD model. Notably, increased expression of the master autophagy regulator Atg1 promotes dAux–Atg9 interaction. Thus, we have identified a dAux–Atg1–Atg9 axis relaying signals through the Atg9 phosphorylation at T62 and T69; these findings further elaborate the mechanism of dAux regulating glial autophagy and highlight the significance of protein degradation pathway in glia contributing to PD.

## Introduction

The patients with Parkinson’s disease (PD) exhibit classical motor symptoms accompanied by non-motor features, progressive degeneration of dopaminergic (DA) neurons, and the formation of Lewy bodies in the brains (Dauer & Przedborski, 2003; Jankovic, 2008; Raza et al., 2019). The underlying mechanism of PD is complex, and it is widely recognized that glial cells play pivotal roles in PD. They contribute by releasing pro- or anti-inflammatory factors to exacerbate or ameliorate disease progression (Cheng et al., 2018; Filippini et al., 2019; Gleichman & Carmichael, 2020; Subhramanyam et al., 2019; Wang et al., 2022) and signaling bidirectionally to regulate neuronal survival and function. Thus, unraveling the glial regulatory mechanism is crucial in advancing our understanding of PD.

**Figure 1.**
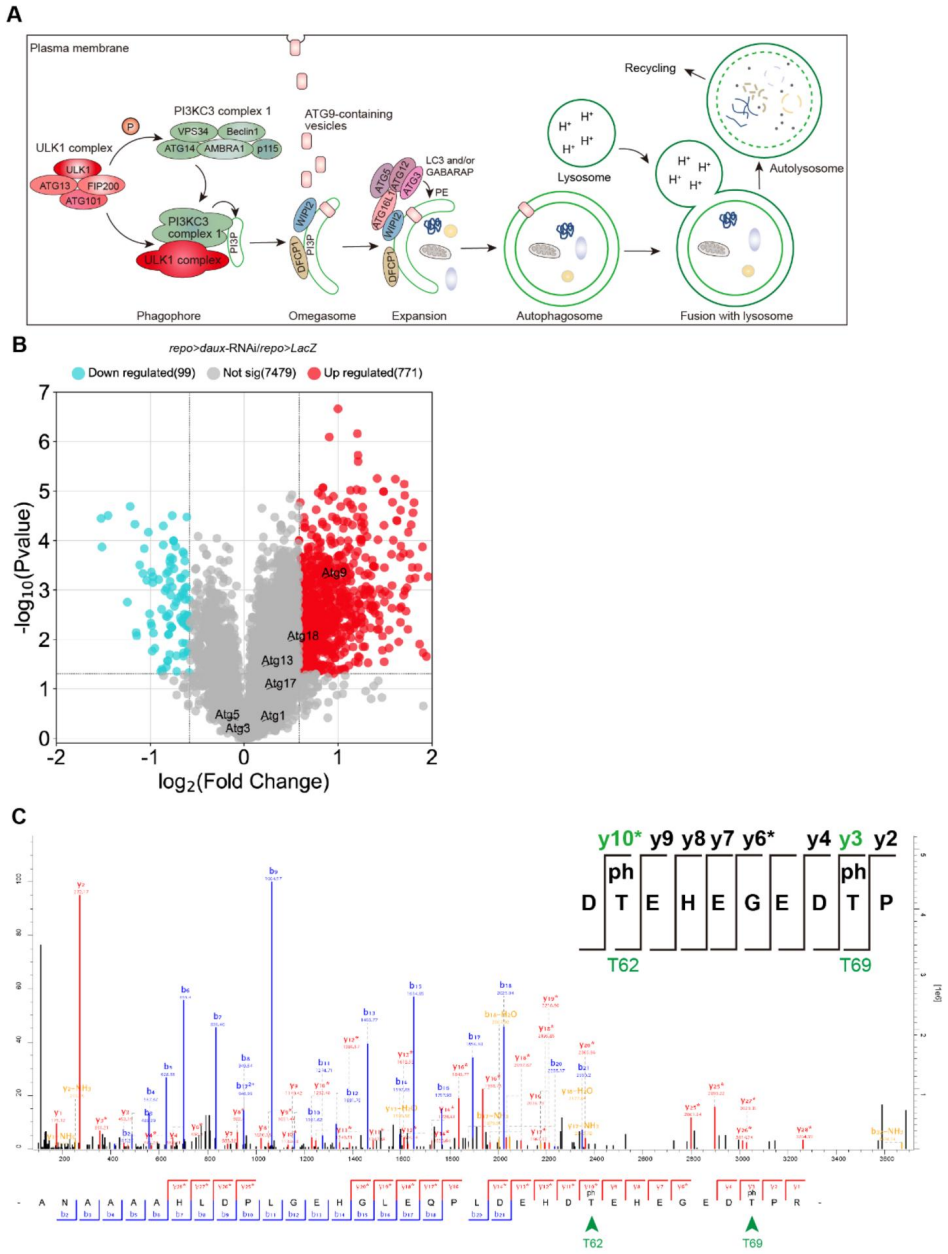
Atg9 phosphorylation at T62 and T69 is upregulated in the absence of glial dAux. (**A**) Illustration of the autophagy process. (**B**) Advanced volcano plot drawn with phosphoproteomic data by the OmicStudio tools. The variance threshold is >1.5 or <0.5, and *p*-value <0.05. (**C**) Phosphoproteomic analysis of the control and *repo > daux-*RNAi adult fly brains. Note that Atg9 phosphorylation at T62 and T69 are detected.

Autophagy is a highly conserved catabolic process to engulf and degrade cytoplasmic materials, and its dysregulation has been linked to many disorders including neurodegenerative diseases (Dikic & Elazar, 2018; Frake et al., 2015; Kim et al., 2017; Levine & Kroemer, 2019; Menzies et al., 2017). The activation of the Unc-51-like kinase 1 (ULK1, homolog of yeast ATG1 and fly Atg1) complex triggers phagophore nucleation by phosphorylating components of the class III PI3K complex (PI3KC3), leading to the local phosphatidylinositol-3-phosphate production and the recruitment of autophagy effectors DFCP1 and WIPI2. WIPI2 promotes LC3 lipidation via the ATG12-ATG5-ATG16L conjugation system for phagophore expansion, which then closes to form a double-membrane autophagosome. Subsequent fusion of autophagosome with the lysosome results in an autolysosome, in which its contents are degraded and the salvaged nutrients are released back into the cytoplasm for recycling (Figure 1A).

Interestingly, phagophore initiation and elongation are facilitated by ATG9, the sole transmembrane protein in the core autophagy machinery for providing lipids as autophagosome membrane sources (Noda, 2017; Ungermann & Reggiori, 2018). It has been shown that ATG9 is transported to the pre-autophagosomal structure and autophagosomes to promote autophagy initiation upon autophagy induction (Mari et al., 2010; Orsi et al., 2012; Reggiori et al., 2004; Yamamoto et al., 2012; Young et al., 2006). ATG9 dysregulation has also been shown in α-syn overexpression or PD animal models (Winslow et al., 2010; Yang et al., 2022; Yi et al., 2024), but how it contributes to PD remains largely unknown.

Here we present evidence that lack of dAuxilin (dAux), the *Drosophila* homolog of the PD risk factor Cyclin G-associated kinase (GAK, also known as DNAJC26), enhances Atg9 phosphorylation at two newly identified threonine resides T62 and T69 as revealed by the phosphoproteomic analysis. The enhanced Atg9 phosphorylation promotes autophagosome formation and Atg9 trafficking to the autophagosomes. Alternatively, the expression of non-phosphorylatable Atg9 variants suppresses the lack of dAux-induced increase in both autophagosome formation and Atg9 autophagic trafficking. The expression of phosphomimetic Atg9 variants restores the lack of Atg1-induced decrease in both events. Moreover, the Atg1 presence enhances dAux–Atg9 interaction. Considering that dAux interacts with Atg1 and Atg9 has been shown as an Atg1 phosphorylation target, our results support the mechanism of a dAux–Atg1–Atg9 axis conveying signal via Atg9 phosphorylation at T62 and T69 for autophagy initiation in adult fly glia. Finally, enhanced Atg9 phosphorylation contributes to progressive DA neuron loss and locomotion deficits in a *Drosophila* PD model, demonstrating its pathological relevance.

## Materials and Methods

### *Drosophila* Genetics

All fly crosses were conducted at 25 °C. Strains are acquired from Bloomington *Drosophila* Stock Center, Vienna *Drosophila* RNAi Center, or used previously: *UAS-LacZ* (BL#1777), *UAS-mCherry.Atg8a* (BL#37750), *UAS-atg9-RNAi* (BL#28055), *UAS-atg1*-RNAi (BL#80434); *UAS-daux-*RNAi (V#16182), *repo-GAL4* (Yang et al., 2022), and *UAS-EGFP.Atg9* (Zhang et al., 2023). Adult flies at 10-day-old were analyzed. Detailed genotypes are listed in Table S1.

### Plasmid Cloning

The DNA sequences expressing Atg9 and Atg9 mutants (Atg9^T62A^, Atg9^T69A^, Atg9^T62A-T69A^, Atg9^T62E^, Atg9^T69E^, and Atg9^T62E-T69E^) were synthetized and then subcloned into *pUAST-attB* vectors containing 3xHA epitope tag by Genscript (Nanjing, China). Fly embryo microinjection was carried out by the *Drosophila* Core Facility, Institute of Biochemistry and Cell Biology, Chinese Academy of Sciences.

### RT-qPCR

Total RNAs of the adult fly heads were extracted by TransZol Up (Cat. #ET111-01, TransGen, Beijing, China) for RT-qPCR analysis using the HiScript III RT SuperMix (Cat. #R323-01, Vazyme, Nanjing, China), the ChamQ Universal SYBR qPCR Master Mix (Cat. #Q711-02, Vazyme), and an ABI 7500 RT-PCR system. The comparative Threshold Cycle (Ct) method was used for quantification. The Ct values were normalized to *rp49*. Relative quantification was performed using the ΔΔCT method.

The primers used are listed below:

*atg1*: described previously (Yan et al., 2019).
*rp49*-F: CCACCAGTCGGATCGATATGC
*rp49*-R: CTCTTGAGAACGCAGGCGACC
*atg9*-F: AGCAGAAGCACGGATTCACA
*atg9*-R: GCAGTGCATCACAAAGGCAA

### Immunohistochemistry

For adult fly brain dissection, adult male flies with the desired genotype were collected in 1.5 mL centrifuge tubes and placed on ice for anesthesia. Several dissection needles, a dissection board, two pairs of dissection forceps, and 1X phosphate buffered saline (PBS) solution were prepared for the dissection procedure, and all these procedures were conducted under a microscope. After adding 1 mL of PBS solution to the dissection board, the anesthetized flies were transferred to the board, and a dissection needle was inserted into the thorax of each fly to fix the fly in the solution. The mouthparts were removed. Forceps were used to peel off the epidermis of the brain gently and remove the eyes, exposing the brains. After that, the flies were placed in a 20% formaldehyde PBS solution for fixation at room temperature for 35 min. Following fixation, the solution was discarded, and the samples were washed three times with 1X PBST (PBS + 0.1% TX-100) solution for 10 min each, after which PBS was added to the samples. Finally, the flies were returned to the dissection board for further dissection to clean up any remaining skulls and fibrous brain tissue. The brains were then blocked in PBST with 5% normal donkey serum, and stained with primary antibodies at 4 °C overnight, and then secondary antibodies at room temperature for 2 h. Primary antibodies used were rat anti-TH (1:300, Cat. #NB300-109, Novus Biologicals, Littleton, CO, USA). The secondary antibodies used were donkey anti-rat Cy3 (1:1000, Cat. #712-165-153, Jackson ImmunoResearch, West Grove, PA, USA).

### Western Blot Analysis

Adult male flies were frozen and stored at –80 °C. Heads were separated from the bodies using vigorous shaking followed by filtering through a sieve, collecting the heads in a grinding tube with lysis buffer (1 µL per head, 0.4% NP-40, 20% glycerol, 0.2 mM EDTA, 100 mM Tris-HCl pH7.5, 150 mM NaCl, 0.5 mM phosphodiesterase inhibitors, 2% Tween 20, and 1 mM PMSF). The samples were then homogenized using a grinding machine or a motorized pestle (Cat. #116005500, MP Biomedicals, Irvine, CA, USA). Following homogenization, the samples were centrifuged at 500 g for 5 min at 4 °C, and the supernatant was discarded. The pellet was washed twice with PBS and added with 100 μL lysis buffer before incubating on a shaking platform at 4 °C for 30 min. The lysate was subsequently centrifuged at 13,000 rpm for 10 min at 4 °C, and the supernatant was collected. Protein concentration was then determined using a BCA protein assay kit, and lysis buffer was added to normalize. Next, proteins were separated on 7.5% SDS-PAGE gels and transferred to PVDF membranes (Cat. #IPFL00010, Millipore, Billerica, MA, USA). For each PAGE gel, two sheets of filter paper (10 cm × 8 cm) and one sheet of PVDF membrane (5 cm × 8.5 cm) were prepared. To prepare the Tris-Glycine buffer for Western Blot transfer, 3.03 g of Tris base and 14.4 g of glycine were dissolved in 800 mL of ddH2O, 200 mL of methanol was added, and the volume was adjusted to 1 L with ddH2O. The solution was stored at 4 °C and used fresh for each Western Blot transfer. The PVDF membrane was activated in methanol for 2 min and subsequently soaked in ddH2O for 2 min. Any excess PAGE gel was trimmed, and the PAGE gel and PVDF membrane were arranged following the black gel–white membrane method before being placed in the transfer apparatus. The setup was run at a constant current of 240 mA for 110 min. The membranes were blocked in 5% fat-free milk in PBST (PBS + 0.1% tween-20) for 40 min. Samples were incubated with the primary antibodies at 4 °C overnight and then with HRP-conjugated secondary antibodies at room temperature for 2 h. Primary antibodies used include: mouse anti-α-Tubulin (1:5000, Cat. #T9026, Sigma), rabbit anti-HA (1:1000, Cat. #3724 T, Cell Signaling Technology, Danvers, MA, USA), rabbit-anti-Myc (1:2000, Cat. #0912-2, Hua An Biotechnology, Hangzhou, China), and mouse anti-Flag (1:1000, Cat. #F3165, Sigma, St. Louis, MO, USA). Secondary antibodies used include: goat anti-mouse-HRP (1:5000, Cat. #115-035-003, Jackson ImmunoResearch, West Grove, PA, USA) and goat anti-rabbit-HRP (1:5000, Cat. #111-036-003, Jackson ImmunoResearch, West Grove, PA, USA). Clarity Western ECL Substrate (Cat. #SB-WB001, Share-bio, Shanghai, China) was used to visualize bands.

### Co-immunoprecipitation

*Drosophila* S2 cells were cultured in Schneider’s Drosophila medium (Cat. #21720024, Gibco, New York, USA) at 28 °C and transfected using the Effectene Transfection Reagent (Cat. #301425, Qiagen, Venlo, The Netherlands) according to the manufacturer’s protocol. Cells were transfected 6 h after seeding and CuSO_4_ was added at a final concentration of 1 mM 24 h after transfection, and cells were harvested after another 24 h. Cells from two 10 cm dishes (about 5 × 10^6^ cells) were lysed on ice using the lysis buffer (0.4% NP-40, 20% glycerol, 0.2 mM EDTA, 100 mM Tris-HCl pH7.5, 150 mM NaCl, 0.5 mM phosphodiesterase inhibitors, 2% Tween 20, and 1 mM PMSF) for 30 min. For pull-downs, the samples were centrifuged at 13,000 rpm at 4 °C for 10 min. Prewashed anti-FLAG(R) M2 beads (Cat. #A2220; Sigma) or rabbit-anti-Myc beads (1:2000, Cat. #0912-2, Hua An Biotechnology, Hangzhou, China) were used to incubate the soluble supernatant at 4 °C overnight. The next day, samples were washed with lysis buffer for 30 min three times. The supernatant was removed, 100 μL of 1X SDS-Dye was added and boiled at 100 °C for 10 min and centrifuged at 13,000 rpm for 5 min at 4 °C and then used for western blot analysis. The mean intensity of the target protein bands were quantified using ImageJ (National Institutes of Health, Bethesda, MD, USA) and data were presented by GraphPad Prism 8 (Dotmatics, Boston, MA, USA).

### Fly Locomotion

The climbing assay was conducted to assess the fly locomotor function as described previously (Cao et al., 2017; Gargano et al., 2005; Song et al., 2017). Yeast-free food was prepared and stored at 4 °C (using the same recipe for normal food but without yeast). *Drosophila* offsprings were collected daily, separated by sex, and allowed to acclimate in yeast-free conditions for at least 12 h before the assay. Both male and female flies were tested. Climbing experiments were performed at desired time points. The climbing apparatus and a DV camera were set up. Ten flies were placed in each tube, with 100 flies prepared for each genotype. Flies were added to the transparent tubes and mounted in the detection apparatus, ensuring each genotype was recorded, and the DV camera was set to capture all tubes at the same level. The device was programmed to vibrate every 30 s, causing the flies to fall to the bottom of the tubes, after which the climbing process was recorded and repeated three times. Video software was used to capture screenshots of the flies’ positions 5 s after falling. A customized software RflyDetection was used to analyze the climbing distances of the flies during this 5-s interval, and the data were recorded. At least three independent experiments were performed.

### Confocal Microscopy and Quantification

Images were acquired by scanning serial Z-stack sections at the similar plane using Nikon C2 or TI2-E + CSU W1 Sora Spinning Disk confocal microscope (20× objective NA = 0.75, 60× oil objective NA = 1.4, Tokyo, Japan). Representative single layer images or maximum projection images are shown. ImageJ (National Institutes of Health, Bethesda, MD, USA) was used to analyze puncta. The intensity threshold was used to quantify numbers of dots. Colocalization plugin, a tool that automatically correlates the two using the intensities, was used for colocalization measurements and the results were shown as Manders’ Correlation (M1 or M2).

### Statistical Analysis

GraphPad Prism 8 (Dotmatics, Boston, MA, USA) was used to present data and analyze significance. Shapiro–Wilk normality test was used to check the normal distribution of data. Two-tailed unpaired *t*-test or ordinary one-way ANOVA followed by Tukey’s multiple comparisons test were used for normally distributed datasets, and Mann–Whitney test or Kruskal–Wallis tests followed by Dunn’s multiple comparisons test were used for datasets without normal distribution. *P*-values <0.05 are considered significant. ns: no significance, *p* ≥ 0.05; *: *p* < 0.05; **: *p* < 0.01; ***: *p* < 0.001; ****: *p* < 0.0001.

## Results

### Atg9 Phosphorylation at T62 and T69 is Upregulated in the Absence of Glial dAux

**Figure 2.**
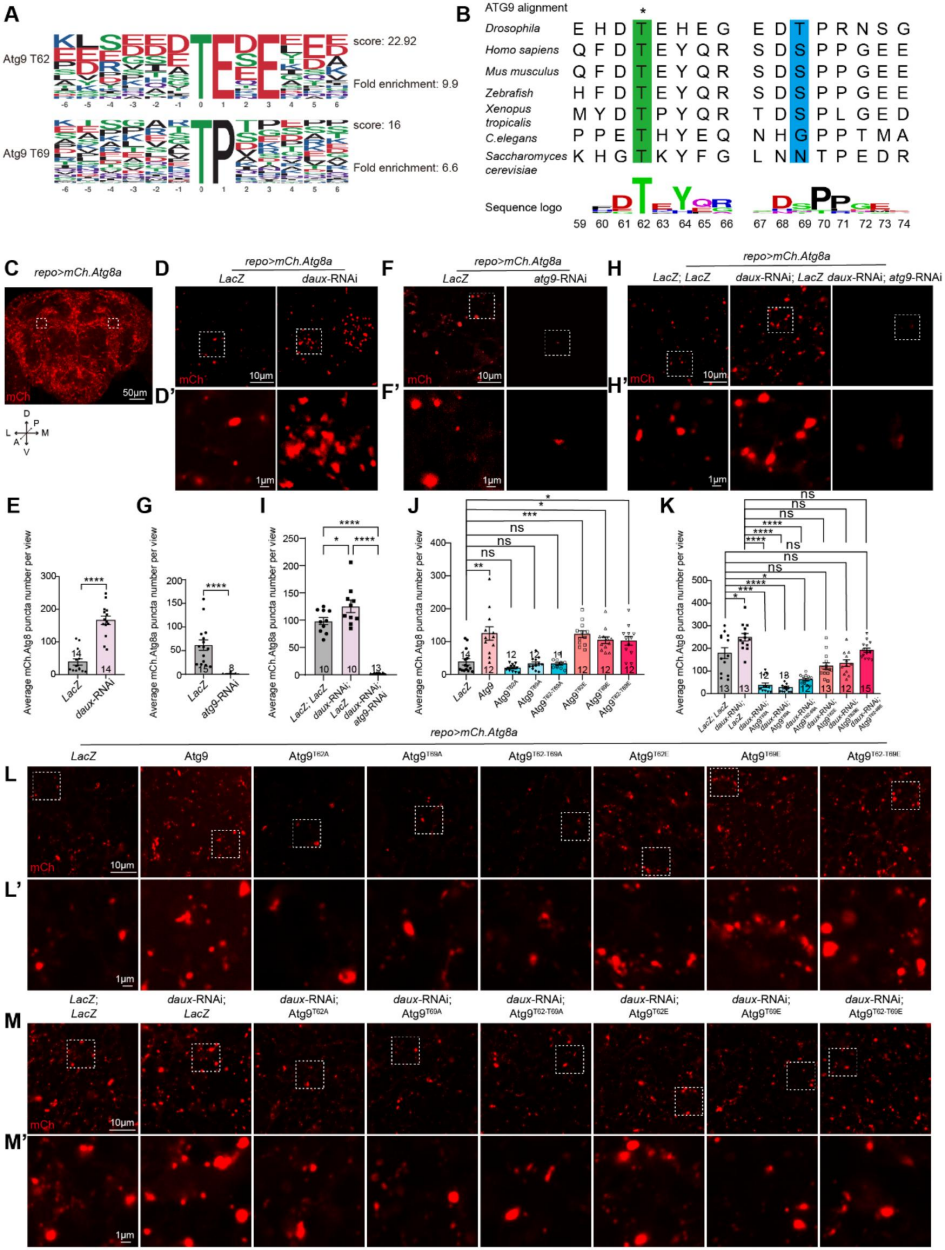
Lack of dAux-enhanced Atg9 phosphorylation at T62 and T69 promotes autophagosome formation in glia. (**A**) The sequences near T62 (xxxxxx_T_ExExxx) and T69 (xxxxxx_T_Pxxxxx) sites of Atg9 belong to phosphorylation motifs by the MoMo analysis tool using the motif-x algorithm. The motif consists of six amino acids upstream and downstream of the potential dAux modification site. Preferred amino acids are indicated with larger font, while less preferred amino acids are indicated with smaller font. (**B**) Alignment of Atg9 protein sequence across different species. Sequence logos are drawn with webLogo. Note that the T62 site is conserved across species. (**C**) An acquired microscopic image of an adult fly brain expressing *UAS-mCherry.Atg8a* under the control of the pan-glial driver *repo*-GAL4 (*repo > mCh.Atg8a*). The adult fly brain is positioned with the coordinates described (A: Anterior, P: Posterior, M: Medial, L: Lateral, D: Dorsal, V: Ventral), with the asymmetrical anterior/dorsal regions enclosed by white dotted squares selected for imaging in all Figures. (**D–I**) Representative images (D, F, and H) and quantifications (E, G, and I) of autophagosomes in adult fly glia. Note that the number of glial autophagosome increases and decreases, respectively, when expressing *daux*-RNAi (D and E) or *atg9*-RNAi (F and G). Expression of *atg9*-RNAi significantly reduced the autophagosome number in the presence of *daux*-RNAi, despite the weak effect of the *daux*-RNAi (H and I). (**J–M**) Representative images (L and M) and quantifications (J and K) of autophagosomes in adult fly glia expressing different Atg9 variants with/without *daux*-RNAi. Note that expressing Atg9 or either of the phosphomimetic Atg9 variants causes a significant increase in the glial autophagosome number, and expressing either of the three non-phosphorylatable Atg9 variants fails to cause significant difference (J and L). Expressing either of the three non-phosphorylatable Atg9 variants suppresses the increased autophagosome number in the absence of glial dAux, whereas expressing either of the three phosphomimetic Atg9 variants fails to reduce the increase significantly (K and M). The areas in white dotted squares in D, F, H, L, and M are enlarged, aligning on the bottom (D’, F’, H’, L’, and M’). Scale bars of different sizes are indicated on the images. Adult male fly brains are dissected for immunostaining and WB analyses. Serial confocal Z-stack sections were taken at similar planes across all genotypes, with representative images shown as a single layer. Statistical graphs are shown with scatter dots indicating the number of brain samples or biological replicates analyzed (*n*, also on the bar). Data are shown as mean ± SEM. *P*-values of significance (indicated with asterisks, ns no significance, *p < 0.05, **p < 0.01, ***p < 0.001, and ****p < 0.0001) are calculated by Mann–Whitney test, ordinary one-way analysis of variance (ANOVA) followed by Tukey’s multiple comparisons test, or Kruskal–Wallis tests followed by Dunn’s multiple comparisons test.

In our previous investigations, we uncovered a dAux-mediated autophagic mechanism in flies and its potential relevance to PD (Wang et al., 2024; Zhang et al., 2023). As we defined a link between dAux and the master regulator Atg1 in autophagy initiation, we sought to explore further how dAux confers its regulation on autophagy via Atg1. Revisiting the reported phosphoproteomic analysis used for detecting changes in phosphorylated peptides in 10-day-old adult fly heads of control and *repo > daux*-RNAi flies (Zhang et al., 2023), we found that phosphorylation at two threonine residues,T62 (*daux*-RNAi/*LacZ* ratio 1.774) and T69 (*daux*-RNAi/*LacZ* ratio 1.774), of the autophagy protein Atg9 (the fly homolog of mammalian ATG9) was significantly upregulated upon glial dAux depletion as shown by the volcano plot and the mass spectrum analysis on the identified Atg9 phosphorylated peptides (Figures 1B and C) (Zhang et al., 2023). Motif analysis predicted that T62 and T69 belong to sequence motifs prone to be phosphorylated namely, xxxxxx_T_ExExxx and xxxxxx_T_Pxxxxx, respectively (Figure 2A). T62, but not T69, is a highly conserved residue across species (Figure 2B), but neither of these two residues has been reported as targets for Atg9 phosphorylation. Given that lack of dAux causes enhanced phosphorylation of these two residues, Atg9 is unlikely to be a direct phosphorylation substrate of dAux.

### Enhanced Atg9 Phosphorylation at T62 and T69 Increases the Autophagosome Formation in Glia

We next sought to determine the physiological significance of Atg9 phosphorylation at T62 and T69. Transgenic flies expressing the Atg9 variants with the threonine residues mutated to either alanine (non-phosphorylatable Atg9^T62A^, Atg9^T69A^, and Atg9^T62A-T69A^) or glutamate (phosphomimetic Atg9^T62E^, Atg9^T69E^, and Atg9^T62E-T69E^) were created. These Atg9 variants were expressed properly in adult male fly brains as examined by WB analysis (Figures S1A and S1B). Taking advantage of these variants, glial autophagosomes in the selected anterior/dorsal glia-rich region of the adult male fly brain were analyzed by expressing the *UAS-mCherry.Atg8a* under the control of a pan-glial driver (*UAS-mCherry.Atg8a; repo-*GAL4, Figures 2C). Consistent with our previous findings, downregulating dAux expression increased the number of the mCherry.Atg8a-labeled autophagosomes in glia (Figures 2D, E, S1C, and S1D). In contrast, *atg9*-RNAi expression, whose efficiency was verified by qRT-PCR (Figure S1E), significantly reduced the autophagosome number in glia (Figures 2F and G). To test Atg9 effect on the *daux*-RNAi-induced increase in autophagosome number, we co-expressed *atg9*-RNAi and *daux*-RNAi in adult fly glia. Despite that expressing two UAS transgenes has titrated out the GAL4 effect, so that the increase was not as obvious as single UAS transgene, we consistently detected an increase in the autophagosome number in *daux*-RNAi-expressing glia (Figures 2D, E, H, I, K, M). *atg9*-RNAi expression suppressed the autophagosome number in the presence of *daux*-RNAi (Figures 2H and I). These results suggest that Atg9 acts downstream of dAux and is important for dAux-mediated autophagosome formation. Consistent to results from *daux*-RNAi, expressing Atg9 or either of the phosphomimetic Atg9 variants caused a significant increase in the glial autophagosome number, and expressing either of the three non-phosphorylatable Atg9 variants failed to cause significant difference (Figures 2J and L). Taken together, these results support the notion that lack of dAux increases Atg9 phosphorylation at T62 and T69, leading to enhanced number of autophagosomes in glia.

### Expression of the Non-Phosphorylatable Atg9 Variants Suppresses the Increased Autophagosome Formation in the Presence of *daux*-RNAi

To further investigate the *in-vivo* relationship between dAux and the Atg9 phosphorylation at these two residues, we co-expressed different Atg9 variants with *daux*-RNAi, to see if the change in Atg9 phosphorylation by these means affects the *daux-*RNAi-induced increase in the autophagosome number. Interestingly, expressing either of the three non-phosphorylatable Atg9 variants suppressed the increased autophagosome number in the presence of *daux*-RNAi, whereas expressing either of the three phosphomimetic Atg9 variants failed to reduce the increase significantly (Figures 2K and M). These results suggest that altered Atg9 phosphorylation levels at T62 and T69 contribute to the dAux-mediated autophagosome formation in glia.

### Atg1 Presence Recruits Atg9 for dAux Interaction

**Figure 3.**
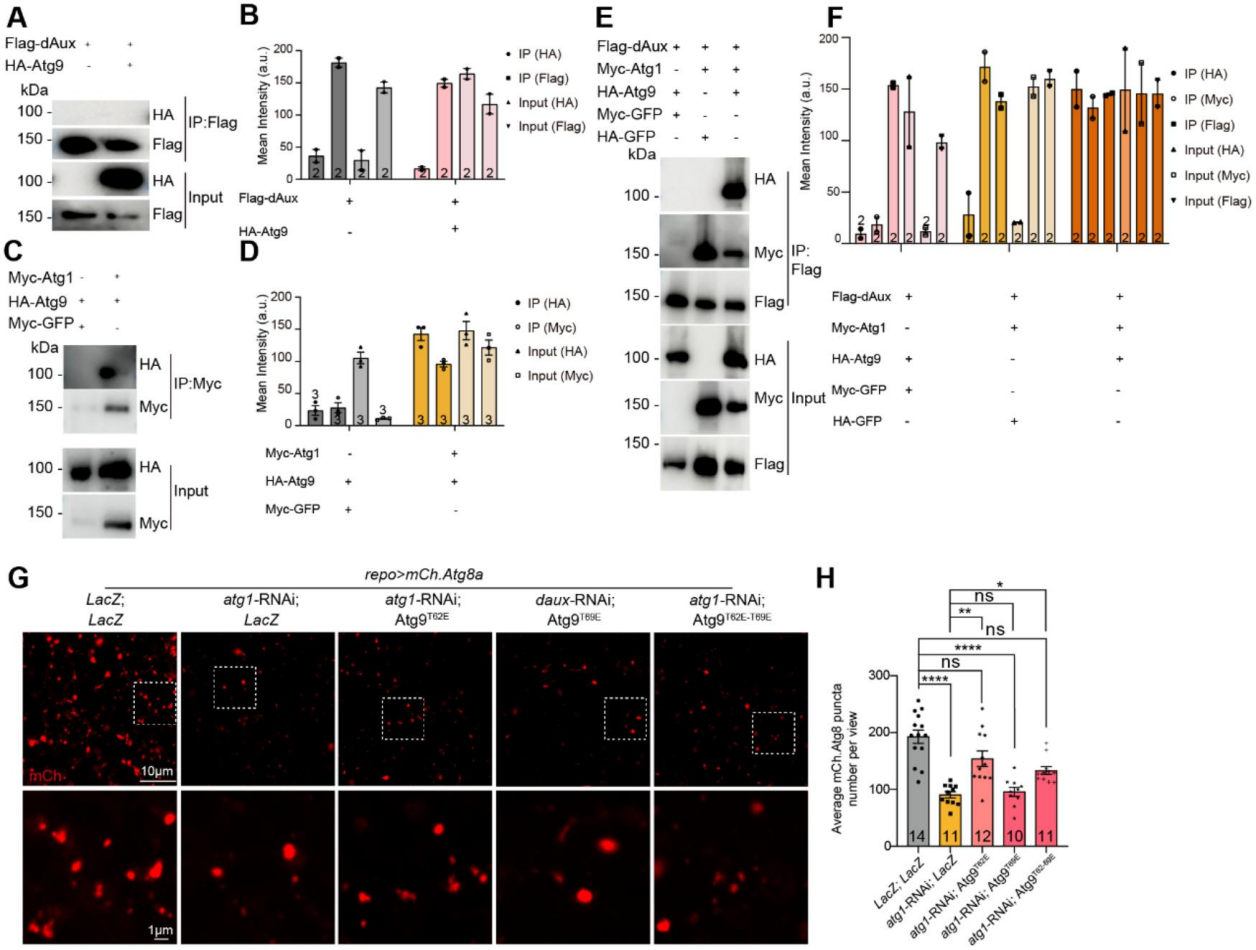
dAux–Atg9 interaction depends on Atg1. (**A**–**F**) Co-IP analysis using the fly S2 cells expressing Flag-dAux, HA-Atg9, or Myc-Atg1 in different combinations was conducted. Note that Atg9 and dAux fail to interact when co-expressing Flag-dAux and HA-Atg9 and detecting with the anti-Flag antibodies (A). Atg1 and Atg9 are within the same complex when using beads conjugated to anti-Myc antibodies to pull down Myc-Atg1 (C). Atg9 is detected in cells expressing Flag-dAux, Myc-Atg1, and HA-Atg9 pulled down by beads conjugated to anti-Flag antibodies (E). Quantifications are shown in B, D, and F. (**G** and **H**) Representative images (D) and quantifications (E) of autophagosomes in adult fly glia expressing different Atg9 variants with *atg1*-RNAi. Note that co-expression of Atg9^T62E^ and Atg9^T62E-T69E^ restores the decreased autophagosome number upon glial Atg1 depletion. Scale bars are indicated on the images. Adult male fly brains are dissected for immunostaining and WB analyses. Serial confocal Z-stack sections were taken at similar planes across all genotypes, with representative images shown as a single layer. Statistical graphs are shown with scatter dots indicating the number of brain samples or biological replicates analyzed (*n*, also on the bar). Data are shown as mean ± SEM. *P*-values of significance (indicated with asterisks, ns no significance, *p < 0.05, **p < 0.01, ***p < 0.001, and ****p < 0.0001) are calculated by Kruskal–Wallis tests followed by Dunn’s multiple comparisons test.

It has been reported that Atg1 mediates Atg9 phosphorylation at multiple sites (Papinski et al., 2014; Reggiori et al., 2004; Zhou et al., 2017). Our previous study also showed that dAux regulates Atg1 trafficking in autophagy (Zhang et al., 2023). Next, we tested if Atg1 is involved in the lack of dAux-enhanced Atg9 phosphorylation. Consistent to previous findings, our co-immunoprecipitation (Co-IP) analysis revealed that Atg1 interacted with Atg9, yet failed to detect Atg9 in the pull-downs of dAux when co-expressing dAux and Atg9, further suggesting that Atg9 is not a direct dAux substrate (Figures 3A–D). Nonetheless, Atg9 was detected in the pull-downs of dAux when co-expressing Atg1 (but not GFP), dAux, and Atg9 (Figures 3E and F). These results suggest that dAux, Atg1, and Atg9 are in the same complex and increased Atg1 expression promotes dAux–Atg9 interaction.

To further demonstrate a role of Atg1 in dAux-regulated Atg9 phosphorylation *in-vivo*, we co-expressed different phosphomimetic Atg9 variants and analyzed their genetic relationship with Atg1. Upon *atg1*-RNAi expression, which efficiently reduced *atg1* expression down to <50%, but not completely (Figure S1F), the number of autophagosomes in glia reduced. Yet, co-expression of Atg9^T62E^ or Atg9^T62E-T69E^, but not Atg9^T69E^, partially and significantly restored the decrease (Figures 3G and H). These results further support the notion that Atg9 phosphorylation at T62 and T69 might contribute to Atg1 function in autophagosome formation.

### dAux- and Atg1-Mediated Atg9 Trafficking in Glia Depends on Atg9 Phosphorylation at T62 and T69

**Figure 4.**
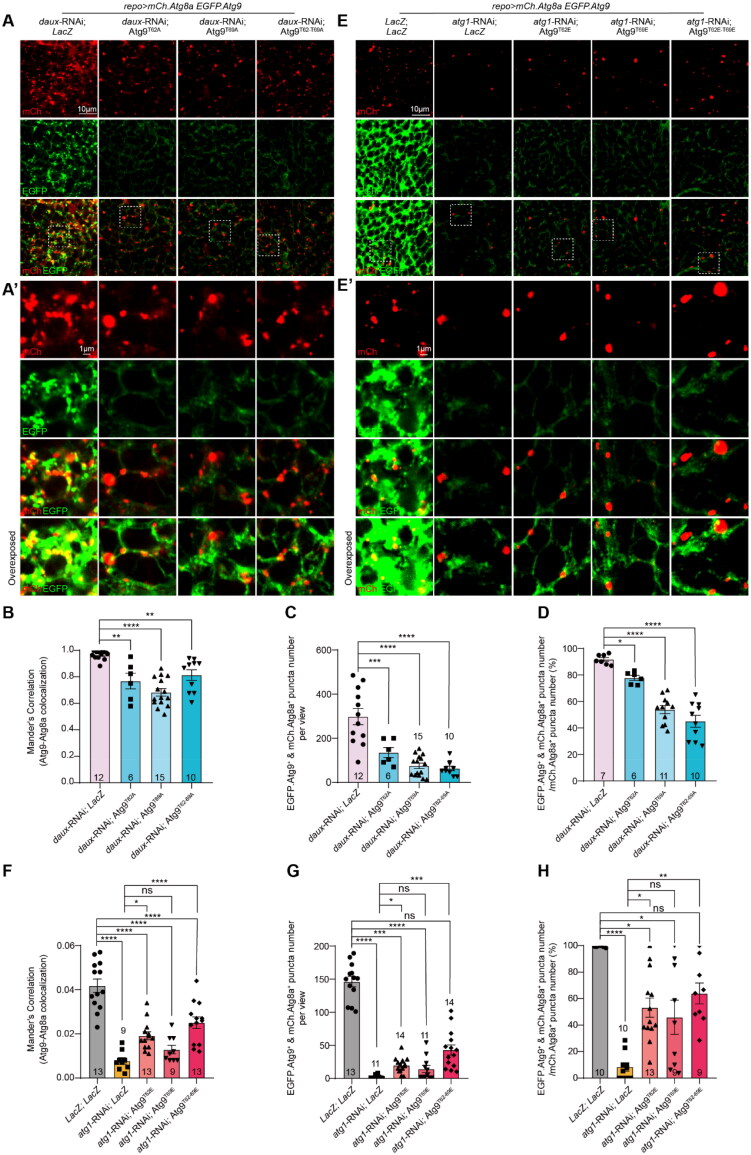
dAux- and Atg1-regulated Atg9 trafficking in glia depends on Atg9 phosphorylation at T62 and T69. (**A**–**H**) Representative images (A and E) and quantifications (B–D and F–H) of Atg9–Atg8a colocalization in adult fly glia expressing different Atg9 variants with *daux*-RNAi or *atg1*-RNAi. *UAS-EGFP.Atg9* and *UAS-mCh.Atg8a* reporters were expressed in glia for analyzing the Atg9 (green)-Atg8a (red) colocalization (*UAS-mCh.Atg8a; repo-*GAL4*, UAS-EGFP.Atg9*). Note that co-expression of either of the three non-phosphorylatable Atg9 mutants partially suppresses the increase of Atg9–Atg8a colocalization upon glial dAux depletion (A), as revealed by the Mander’s Correlation value (B), the number of Atg9 and Atg8a co-positive puncta (C), and the percentage of number of co-positive puncta over the total Atg8a-positive puncta (D). The Atg9–Atg8a colocalization decreases when expressing *atg1-*RNAi, which is restored by expressing Atg9^T62E^ or Atg9^T62E-T69E^ (E and F–H). Areas enclosed by the white dashed squares in the representative images (A and E) are enlarged in A’ and E’. An overexposed panel is listed at the bottom of A’ and E’ for better visualization of signal colocalization. Scale bars of different sizes are indicated on the images. Adult male fly brains are dissected for immunostaining and WB analyses. Serial confocal Z-stack sections were taken at similar planes across all genotypes, with representative images shown as a single layer. Colocalization is analyzed using the Manders’ Correlation, taking into account the change in the protein level. Statistical graphs are shown with scatter dots indicating the number of brain samples or biological replicates analyzed (*n*, also on the bar). Data are shown as mean ± SEM. *P*-values of significance (indicated with asterisks, ns no significance, *p < 0.05, **p < 0.01, ***p < 0.001, and ****p < 0.0001) are calculated by ordinary one-way ANOVA followed by Tukey’s multiple comparisons test and Kruskal–Wallis tests followed by Dunn’s multiple comparisons test.

It has been shown that Atg9 phosphorylation is involved in its trafficking to the autophagy initiation site (Feng et al., 2016; Mack et al., 2012; Zhou et al., 2017). We next tested whether Atg9 phosphorylation at T62 and T69, like other residues, is also important for its trafficking to autophagosomes. Consistent with the results from *daux*-RNAi, expression of Atg9 and the phosphomimetic Atg9^T62E-T69E^ increased the Atg9–Atg8a colocalization, as revealed by the dual expression of *UAS-mCherry.Atg8a* and *UAS-EGFP.Atg9* reporters in glia (Figures S2A and S2B). A similar increasing trend was observed for Atg9^T62E^ and Atg9^T69E^. On the contrary, the expression of non-phosphorylatable Atg9^T62A^ reduced Atg9-Atg8a colocalization significantly, and a similar decreasing trend was observed for Atg9^T69A^ and Atg9^T62A-T69A^ (Figures S2A and S2B). These results indicate that Atg9 trafficking to the autophagosomes is affected by Atg9 phosphorylation at T62 and T69. Notably, co-expressing either of the three non-phosphorylatable Atg9 mutants suppressed the Atg9–Atg8a colocalization in the presence of *daux*-RNAi, as revealed by the Mander’s Correlation value, the number of Atg9 and Atg8a co-positive puncta, and the percentage of number of co-positive puncta over the total Atg8a-positive puncta (Figures 4A–D). These results demonstrate the importance of Atg9 phosphorylation at T62 and T69 in dAux-mediated Atg9 trafficking. Based on the results that increased Atg1 expression promotes the dAux–Atg9 interaction, and also that dAux regulates Atg1 trafficking, we also tested if Atg1 is involved in the dAux-mediated Atg9 trafficking to the autophagosomes. Whereas *atg1*-RNAi expression in glia blocked Atg9 trafficking to autophagosomes, co-expression of the phosphomimetic Atg9^T62E^ or Atg9^T62E-T69E^ partially and significantly restores the suppression, with the double variants exhibiting a more potent effect (Figures 4E–H). Taken together, our results suggest that Atg9 phosphorylation at T62 and T69 contributes to the dAux- and Atg1-regulated Atg9 trafficking to the autophagosomes.

### Enhanced Atg9 Phosphorylation at T62 and T69 Contributes to DA Neurodegeneration and Locomotor Dysfunction

**Figure 5.**
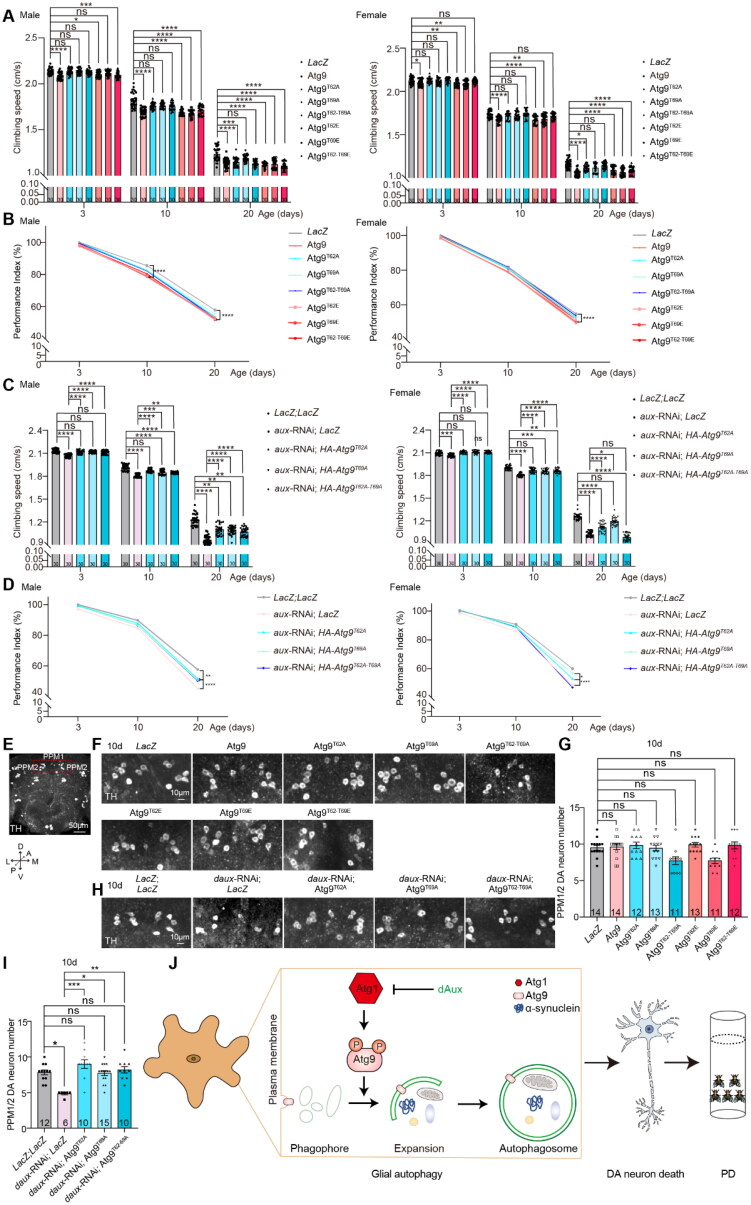
Enhanced Atg9 phosphorylation at T62 and T69 contributes to DA neurodegeneration and locomotor dysfunction. (**A**–**D**) Locomotor activity of 3-, 10-, and 20-day-old adult male and female flies is analyzed. Note that expression of Atg9 or either of the three phosphomimetic Atg9 variants results in a significant reduction in the climbing speed and performance index of adult male and female flies, while the expression of the non-phosphorylatable Atg9 variants fails to cause significant difference (A and B). Co-expression of *daux*-RNAi with either of the three non-phosphorylatable Atg9 mutants restores the *daux*-RNAi-induced climbing speed and performance index deficits in both male and female flies (C and D). The performance index indicates the relative decline of the climbing ability between aged and young (3-day-old) flies in a climbing assay: performance index = velocity_aged_/velocity_young_. (**E**) An acquired microscopic image of an adult fly brain stained with anti-TH antibodies revealing all DA neuron clusters, the protocerebral posterior medial (PPM)1 and PPM2 clusters enclosed by red dotted squares are selected for further analyzing. The adult fly brain is positioned with the coordinates described (A: Anterior, P: Posterior, M: Medial, L: Lateral, D: Dorsal, V: Ventral). (**F**–**I**) Representative images (F and H) and quantifications (G and I) of DA neuron number in 10-day-old adult fly brains with the indicated genotypes. Note that expression of either of the three non-phosphorylatable or phosphomimetic Atg9 variants fails to cause significant changes in the DA neuron number at the PPM1/2 cluster. Co-expressing either of the three non-phosphorylatable Atg9 mutants rescues the DA neuron loss upon glial dAux depletion. (**J**) The schematic diagram illustrating a dAux–Atg1–Atg9 axis regulating glial autophagy in PD. Scale bars of different sizes are indicated on the images. Both adult male and female flies are tested. Serial confocal Z-stack sections were taken at similar planes across all genotypes, with representative images shown as maximal projection. Statistical graphs are shown with scatter dots indicating the number of brain samples or biological replicates analyzed (*n*, also on the bar). In locomotion experiments, *n* = 100 for each genotype. Data are shown as mean ± SEM. *P*-values of significance (indicated with asterisks, ns no significance, * *p* < 0.05, ** *p* < 0.01, *** *p* < 0.001, and **** *p* < 0.0001) are calculated by ordinary one-way ANOVA followed by Tukey’s multiple comparisons test or Kruskal–Wallis tests followed by Dunn’s multiple comparisons test.

As Atg9 dysregulation has been implicated in PD (Winslow et al., 2010; Yang et al., 2022; Yi et al., 2024), we tested whether Atg9 phosphorylation at T62 and T69 contributes to locomotor function and DA neurodegeneration in flies. Interestingly, expression of Atg9, or either of the three phosphomimetic Atg9 variants, resulted in a significant reduction in the climbing speed and performance index of 3-, 10-, and 20-day-old adult male flies, with the exception for Atg9^T69E^ at day 3. Female flies exhibited a similar trend, with most severe decline at day 20 (Figures 5A and B). The expression of the non-phosphorylatable Atg9 variants failed to cause significant difference until day 20, seen more significantly in male flies (Figures 5A and B). Intriguingly, co-expression of *daux*-RNAi with either of the three non-phosphorylatable Atg9 variants restored the *daux*-RNAi-induced locomotor deficits in 3-, 10-, and 20-day-old adult male and female flies, with a weak effect for females at day 20 (Figures 5C and D). These results suggest that dAux and Atg9 phosphorylation at T62 and T69 impact on fly climbing ability, potentially reflecting the pathological locomotor deficits.

Next, DA neurodegeneration in the 10-day-old adult fly brains was analyzed. Expressing either of the three non-phosphorylatable or phosphomimetic Atg9 variants failed to cause significant changes in the DA neuron number at the PPM1/2 cluster, where DA neuron loss has been observed upon α-synuclein overexpression in flies (Feany & Bender, 2000) (Figures 5E–G). Nonetheless, *daux*-RNAi-induced DA neurodegeneration was significantly suppressed upon co-expression of either of the three non-phosphorylatable Atg9 variants (Figures 5H and I). These results suggest that lack of dAux-enhanced Atg9 phosphorylation at T62 and T69 contributes to DA neurodegeneration in adult fly brains.

## Discussion

In the present study, we reported that the phosphorylation of two newly identified Atg9 residues contributes to glial autophagy in a *Drosophila* PD model by analyzing locomotor function and DA neurodegeneration (Figure 5J). Previously, we unraveled the mechanism of a potential PD risk factor, dAux, in autophagy initiation in adult fly glia (Zhang et al., 2023). As an extension of our study, we now further elucidated that Atg9 phosphorylation regulates autophagosome formation and Atg9 trafficking to the autophagosomes in adult fly glia, events important for autophagy initiation. Changes in Atg9 phosphorylation level at T62 and T69 affect the *daux*-RNAi-induced changes in the autophagosome number and the Atg9–Atg8a colocalization, suggesting a potential link between dAux and Atg9 phosphorylation. Considering that Atg1 has been shown to phosphorylate Atg9 at multiple residues, increased Atg1 expression promotes dAux–Atg9 interaction, and dAux regulates Atg1 activity during autophagy initiation, we speculate that dAux regulate Atg9 phosphorylation via Atg1; the dAux–Atg1–Atg9 axis plays a role in the autophagy initiation in adult fly glia. It has been shown that sex differences influence the outcome of experimental results. Given that our immunostaining and WB results are all extracted from adult male flies, it is hard to conclude whether there is any difference in results of either sex. Intriguingly, our climbing results revealed a potential difference in male and female flies. It is essential to consider the sex difference and interpret these behavioral results with caution.

On the other hand, the dAux–Atg1–Atg9 axis might be physiologically and pathologically relevant. Altered Atg9 phosphorylation at T62 and T69 causes changes in fly climbing ability, possibly reflecting motor dysfunction. Lack of dAux-induced locomotor deficits is restored upon changing Atg9 phosphorylation, suggesting that Atg9 phosphorylation acts downstream of dAux in regulating the fly locomotor activity. Interestingly, as lack of dAux induces DA neurodegeneration, loss of these cells is significantly suppressed by expressing Atg9 variants with altered phosphorylation at T62 and T69. Taken together, these results suggest that the identified dAux–Atg1–Atg9 is potentially physiologically relevant. Moreover, locomotor function and DA neurodegeneration are two key hallmarks in PD. Considering our previously proposed role of dAux in a *Drosophila* PD model, and that the mammalian dAux homolg GAK has been shown to be a potential PD risk factor, we speculate that Atg9 phosphorylation acting downstream of dAux, a glial mechanism identified in the present study, might be important for pathological features implicated in PD. These findings underscore the need for further investigation of glial regulation in neurodegenerative diseases such as PD.

## Supplementary Material

Fig S2.pdf

Fig S1.pdf
