# Supplementary figures and images for: New Atg9 Phosphorylation Sites Regulate Autophagic Trafficking in Glia
Source: ASN Neuro. 2025 Jan 14;17(1):2443442. doi: 10.1080/17590914.2024.2443442 (PMC11877618; doi:10.1080/17590914.2024.2443442)

Figure S2

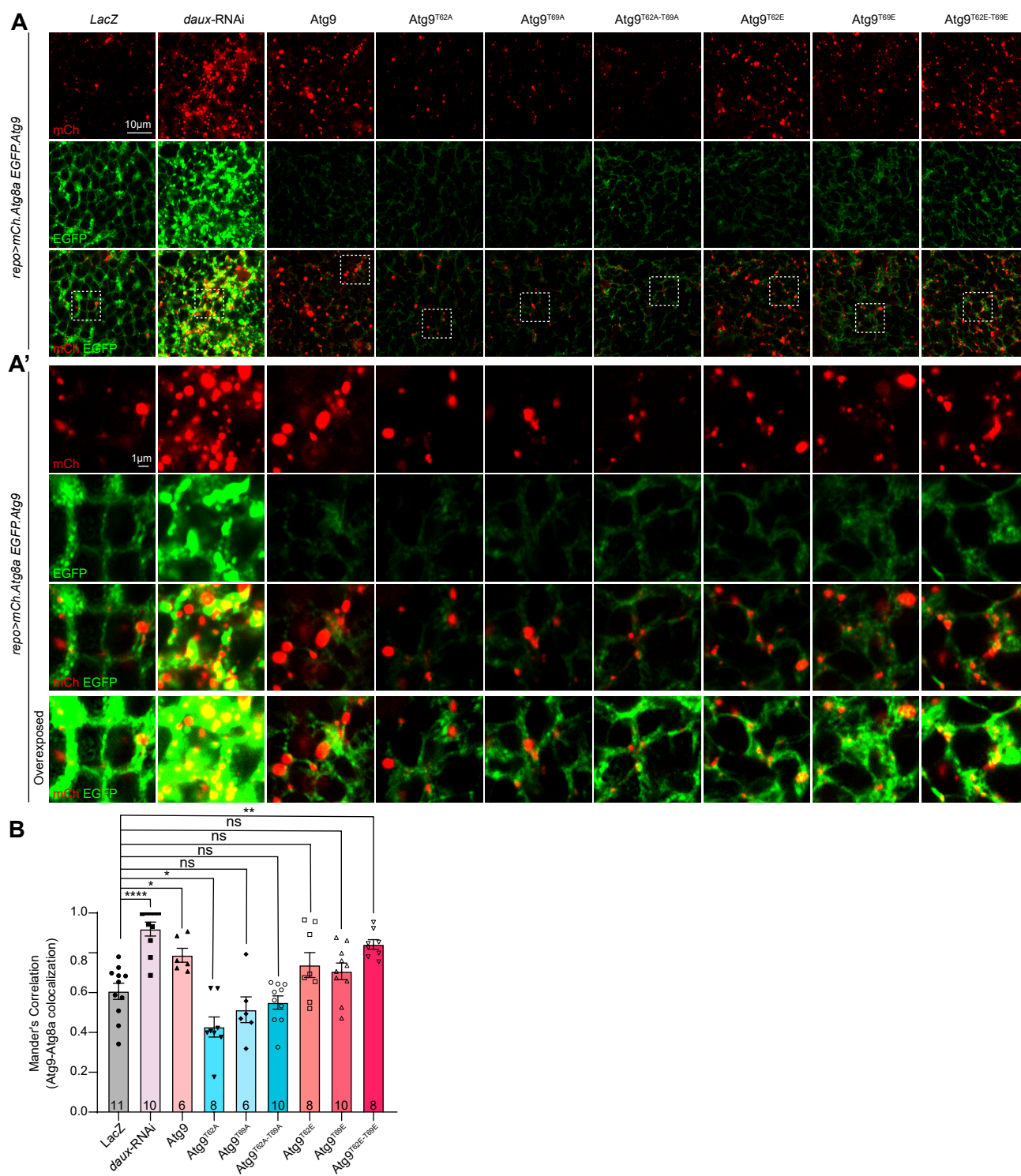

Supplement: Fig S2.pdf [file TASN_A_2443442_SM5563.pdf]

Figure S1

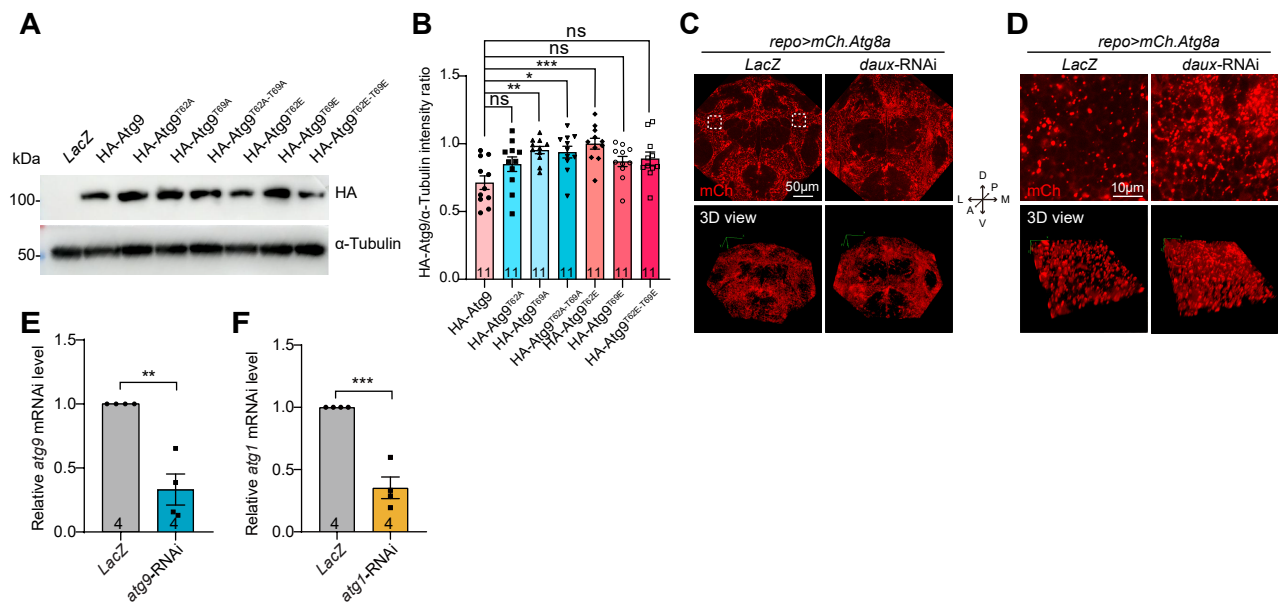

Supplement: Fig S1.pdf [file TASN_A_2443442_SM5562.pdf]
